# Impact of adverse childhood experiences and fruit and vegetable intake in adulthood

**DOI:** 10.1017/S1368980019004932

**Published:** 2021-04

**Authors:** Masako Horino, Wei Yang

**Affiliations:** 1 Department of Epidemiology, Johns Hopkins Bloomberg School of Public Health, Baltimore, MD 20205, USA; 2 Department of Environmental Sciences, University of Nevada, Reno, Reno, NV 89557, USA; 3 Department of Social and Preventive Epidemiology, Division of Health Sciences and Nursing, Graduate School of Medicine, University of Tokyo, Tokyo 113-0033, Japan; 4 United Nations Relief and Works Agency for the Palestine Refugees in the Near East, Jordan, Amman; 5 School of Community Health Sciences, Division of Epidemiology, Biostatistics, and Environmental Health, University of Nevada, Reno, Reno, NV 89557, USA

**Keywords:** Adverse childhood experience, Fruit and vegetable, Epidemiology, Population-based study

## Abstract

**Objective:**

To assess the association between adverse childhood experiences (ACE) and behaviours of fruit and vegetable consumption among adults.

**Design:**

Cross-sectional analysis. Weighted *χ*^2^ and weighted multiple logistic regression analyses were conducted to determine the association between ACE and low fruit and vegetable consumption.

**Setting:**

The 2017 Nevada Behavioral Risk Factor Surveillance System.

**Participants:**

The sample consisted of 2939 adults.

**Results:**

After controlling for potential confounders, exposure to three or more ACE (adjusted OR (AOR) 1·42, 95 % CI 1·02, 2·00) and experiencing parental divorce/separation (AOR 1·50, 95 % CI 1·13, 1·98) were significantly associated with low fruit and vegetable consumption. The study did not find a dose–response relationship between the number of ACE and fruit and vegetable consumption.

**Conclusions:**

The study suggests that participants who experienced three or more ACE or parental divorce/separation were at increased risk for low fruit and vegetable consumption. The findings highlight the continuing need for public health interventions and policies that decrease exposure to ACE and increase fruit and vegetable intake among the populations with ACE.

Adverse childhood experiences (ACE) have been strongly linked to a number of chronic diseases, including CVD, cancer and type 2 diabetes, in adulthood and premature death^(1–5)^. ACE refers a range of traumatic experiences such as abuse and household dysfunction that have occurred prior to the age of 18 years, which pose a substantial risk for the subsequent development of negative health outcomes. ACE is common in the United States, where it was estimated that more than half (61·55 %) of adults are exposed to at least one ACE, and more than one in five adults (24·64 %) have experienced three or more ACE^(6)^.

In recent decades, increasing attention has focused on elucidating the mechanisms that underlie the associations between ACE and chronic diseases in adulthood. During early childhood, the brain undergoes its most rapid period of growth and development and is a highly sensitive stage for the detrimental effects of ACE^(7)^. Without adequate protective factors (e.g. secure attachment and key buffering relationships with caregivers), ACE and other types of childhood adversity, such as poverty, food insecurity, poor educational opportunities and community violence, could lead to a type of stress response known as toxic stress^(8,9)^. Toxic stress causes long-term changes in brain architecture and organ systems. For example, toxic stress generated upon exposure to ACE could lead to biological modifications, such as alterations in hormonal and immune system factors, which may ‘rewire’ the brain so as to make individuals more vulnerable to subsequent stressors^(10)^. Altered patterns of brain development among children exposed to ACE might contribute to the onset of chronic diseases, in part, by increasing the tendency to engage in high-risk behaviours^(9)^. As a matter of fact, ACE is reported to be associated with higher levels of substance use, poor mental health, overeating and obesity^(11,12)^.

Suboptimal diet is a leading preventable risk factor for chronic diseases and has been linked to nearly half of all estimated annual mortality from CVD and diabetes in the United States^(13)^. Regardless of continuous efforts to promote fruit and vegetable intake in the United States, inadequate consumption of these persists. Previous studies have reported that 76 % of adults did not meet fruit intake recommendation (1·5–2·0 cups equivalent of fruits daily), and 87 % did not meet vegetable intake recommendation (2·0–3·0 cups of vegetables daily)^(14,15)^.

Limited cross-sectional research has explored the association between ACE and dietary behaviour. Given the high prevalence of chronic diseases and ACE, a better understanding of these potential relationships, particularly fruit and vegetable intake, has a potential to contribute to public health by highlighting novel targets for intervention^(6)^. Understanding the factors that place groups at a greater risk for poor outcomes could inform the development of effective prevention and intervention strategies, and would ensure that resources are used efficiently to address the needs of vulnerable groups. To address the issue, the present study aimed to extend the literature on ACE and unhealthy behaviour by focusing on low fruit and vegetable intake among adults based on recent population-based data. The study tested the following hypotheses: there is a graded relationship between the number of ACE and low fruit and vegetable intake among adults. In addition, we conducted an exploratory analysis to examine whether any individual types of ACE are associated with low fruit and vegetable intake. Although literature suggests that ACE types seldom occur in isolation, the impacts of individual ACE types were examined since there is no previous literature addressing the relationships between ACE and dietary pattern^(5)^.

## Methods

### Data sources

This cross-sectional study used data from the 2017 Nevada Behavioral Risk Factor Surveillance System (BRFSS). BRFSS is a combined landline and cell-phone health survey that uses a random digit dialling technique^(16,17)^. This telephone survey collects health information among non-institutionalised adults aged ≥18 years. Trained interviewers administered standardised national survey questions, optional questions and state-added questions, including ACE module questions^(16,17)^. In 2017, 3764 Nevadans participated in the BRFSS. Of these, 2939 participants had complete data for all ACE questions.

### Measures

The independent variables of interest were measures of ACE reported by the BRFSS ACE module, which consists of eleven questions adopted from the original ACE study and assessed the occurrence of eight different areas of abuse and household dysfunction prior to the age of 18^(1)^. Measures of abuse included physical abuse (one item), sexual abuse (three items) and psychological/emotional abuse (one item). Measures of household dysfunction included living with an adult who was depressed, mentally ill or suicidal (one item), living with anyone who abused substances (two items), living with someone who had been sentenced to serve time in a prison, jail or other correctional facility (one item), having parents who were separated/divorced (one item), and living in a home where adults or parents physically harmed each other (one item)^(1)^. Items about mental illness, drinking problem, drug use, family incarceration and parental divorce/separation asked whether or not the respondent had been exposed to the respective ACE. The remaining items allowed respondents to report the frequency of occurrence of a specific ACE (i.e. never, once or more than once). For the current study, all items were further dichotomised to indicate whether or not the experience had ever happened. Because three items measured facets of sexual abuse and two items measured parts of living with a substance abuser, they were each combined into a single measure of sexual abuse and a single measure of living with a substance abuser, respectively, resulting in a total of eight ACE measures^(18)^. To assess the cumulative exposure to ACE, the counts of ACE types for each participant were categorised into the following groups: no ACE, 1 ACE, 2 ACE, or ≥3 ACE^(18)^.

Our dependent variable was the frequency of fruit and vegetable intake per day. Fruit and vegetable consumption frequencies were measured by six questions in the BRFSS core module: (i) ‘How often did you drink 100 % fruit juice such as apple or orange juice? Do not include fruit-flavoured drinks or fruit juices you added sugar to.’ (ii) ‘During the past month, how often did you eat fruit? Do not include juices. You can tell me times per day, per week or per month.’ (iii) ‘How often did you eat a green leafy or lettuce salad, with or without other vegetables?’ (iv) ‘How often did you eat any kind of fried potatoes, including french-fries, home fries or hash browns?’ (v) ‘How often did you eat any other types of potatoes, such as baked, boiled, mashed potatoes or potato salad?’ (vi) ‘Not including lettuce salads and potatoes, how often did you eat other vegetables?’ We limited our analyses to fruits and excluded 100 % fruit juice, because fruit juice is associated with an increased risk of type 2 diabetes and greater weight gain^(19,20)^. French-fries and potato consumption was also excluded because three different cohorts of health professionals observed an increased risk for obesity when consuming more potatoes, and a decreased risk for obesity when consuming whole fruits and non-starchy vegetables^(20)^. Responses for fruits, dark green vegetables and other vegetables were converted into values representing consumption per day. The daily consumption values were summed to create a total daily fruit and vegetable consumption variable. Low fruit and vegetable consumption was defined as consuming <2 fruits and/or vegetables daily^(21)^.

Several variables were included as potential confounders in the analyses. Gender was dichotomised into male or female. Age groups were categorised into three groups: 18–34, 35–64, and ≥65. Race included white, black, Hispanic or other. Due to small proportions of Asians, American Indians or Alaskan Natives, and Pacific Islanders, these race categories were combined to other. Marital status was dichotomised into single or married. Educational attainment was described as: high school or less, some college and college graduate. BMI (kg/m^2^) was calculated from self-reported weight and height, and was categorised into three groups: <25, 25–29·9, ≥30·0 kg/m^2^. Poor mental health was assessed by the following BRFSS question: ‘Now thinking about your mental health, which includes stress, depression and problems with emotions, for how many days during the past 30 d was your mental health not good?’ Participants who reported fourteen or greater poor mental health days in the past 30 days were defined as having poor mental health. Previous studies indicated that health risk behaviours often co-occur together in a population^(22)^. Three health risk behaviours (heavy drinking, smoking and physical inactivity) that are well established to be related to dietary quality were chosen as covariates. Participants were categorised as a heavy drinker if they reported more than fourteen drinks per week for males and more than seven drinks per week for females. As for cigarette smoking, participants were asked whether or not they currently smoked every day, some days or not at all. Based on this question, participants who reported they did not currently smoke were defined as non-smokers. All others were defined as current smokers. Leisure-time physical inactivity was dichotomised based on participants’ response when asked if they exercised any, in addition to physical activity performed as part of their employment within the last 30 d.

### Statistical analyses

All analyses were completed using a statistical software (SAS, version 9.4; SAS Institute Inc.). To account for BRFSS sampling methodology, our analyses used survey sampling weights. In descriptive analyses, we reported frequencies and proportions for categorical variables, and means and standard deviations for continuous variables. The weighted *χ* ^2^ test was used to assess the relationship between low fruit and vegetable intake and gender, age, race/ethnicity, marital status, education, smoking status, BMI, poor mental health, heavy drinking, leisure-time physical inactivity, individual ACE types and overall ACE scores. Weighted multiple logistic regression models were used to estimate OR, adjusted OR (AOR) and 95 % CI for the examination of associations between ACE and low fruit and vegetable intake. We conducted two sets of multiple logistic regression analyses controlling for selected covariates. The first set assessed the relationship between cumulative ACE scores and low fruit and vegetable intake, and the second set examined the correlation between each of the eight ACE types and low fruit and vegetable intake. We used bivariate analyses to detect statistical differences between variables and considered *α* = 0·05 as the level of significance.

## Results

Table 1 shows the characteristics of all respondents. Slightly over half of respondents were female (55·5 %), white (53·0 %), single (52·1 %) and aged between 35 and 64 years (50·9 %). 46·1 % of respondents obtained a high school education or less, and 40·1 % of them were categorised into BMI 25–29·9 kg/m^2^. The prevalence of three health risk behaviours was 6·2 % for heavy drinkers, 29 % of leisure-time physical inactivity and 17 % for current smokers. Approximately one-third of respondents experienced parental divorce/separation (33·6 %), verbal abuse (33·0 %) and living with a substance abuser (31·3 %). Nearly two-thirds (65·5 %) of all respondents had experienced at least one ACE, and 28·3 % of all respondents were exposed to ≥3 ACE. 47·2 % of respondents reported low fruit and vegetable intake.

**Table 1:** Sample characteristics, adverse childhood experiences (ACE) and low fruit and vegetable intake, Nevada Behavioral Risk Factor Surveillance System 2017, adults ≥18 years (*n* 2939)

Variables	All respondents	
	*n*	%
Total	2939	100
Gender		
Female	1632	55·5
Male	1308	44·5
Age (years)		
18–34	451	28·1
35–64	1354	50·9
65+	1134	21·1
Race		
White	2083	53·0
Black	120	7·5
Hispanic	444	27·2
Other	248	12·3
Marital status		
Single	1494	52·1
Married	1428	47·9
Highest education		
High school or less	939	46·1
Some college	1013	33·7
College graduate	983	20·2
Current smoker		
Yes	441	18·1
No	2475	81·9
BMI category (kg/m^2^)		
<25	983	33·3
25–29·9	1067	40·1
≥30	728	26·6
Poor mental health (>14 d)		
Yes	328	11·3
No	2580	88·7
Heavy drinker		
Yes	206	6·3
No	2692	93·7
Leisure-time physical inactivity		
Yes	758	29·2
No	2181	70·8
Types of ACE		
Lived with someone who was mentally ill		
Yes	506	15·7
No	2457	84·3
Lived with a substance abuser		
Yes	942	31·3
No	2021	68·7
Family member incarceration		
Yes	216	9·7
No	2747	90·3
Parents divorced/separated		
Yes	914	33·6
No	2049	66·4
Domestic violence in household		
Yes	533	19·0
No	2430	81·0
Physical abuse		
Yes	601	21·3
No	2362	78·7
Verbal abuse		
Yes	1011	33·0
No	1952	67·0
Sexual abuse		
Yes	415	12·3
No	2548	87·7
Number of ACE		
0	1034	34·5
1	692	23·8
2	405	13·6
3+	832	28·3
Low fruit and vegetable intake*		
Yes	1179	47·2
No	1760	52·8

* Consumed fruits, dark green vegetables and other vegetables less than twice a day.

Table 2 presents the weighted prevalence and AOR of low fruit and vegetable intake by covariates and ACE score. Participants who consumed less fruits and vegetables were more likely to be male (55·0 *v*. 39·2 %), single (52·8 *v*. 40·8 %), have high school or less education (high school or less = 51·7 %, some college = 49·2 %, college graduate = 33·8 %), current smokers (59·1 *v*. 44·7 %), have a BMI ≥ 30·0 kg/m^2^ (BMI ≥30·0 kg/m^2^ = 52·4 %; BMI 25·0 to <30·0 kg/m^2^ = 47·7 %; BMI <25 kg/m^2^ = 42·5 %), have poor mental health status (60·7 % *v*. 45·3 %) and did not engage in leisure-time physical activity (59·6 *v*. 42·2 %). The number of ACE did not reach statistical significance based on a *P*-value of 0·71.

**Table 2:** Weighted prevalence of low fruit and vegetable consumption and adjusted OR for the association between adverse childhood experiences (ACE) and fruit and vegetable intake, Nevada Behavioral Risk Factor Surveillance System 2017, adults ≥18 years (*n* 2939)

Variables	Low intake of fruits and vegetables*					
	*n*	%	*P*-value	Adjusted OR†	95 % CI	*P*-value
Total	1179	47·2	0·054			
Gender						
Female (ref)	554	39·2	<0·0001	1·00	–	
Male	624	55·0		1·90	1·47, 2·45	<0·0001
Age (years)						
18–34 (ref)	204	50·6	0·306	1·00	–	
35–64	524	46·1		1·03	0·73, 1·45	0·886
65+	451	45·5		1·16	0·80, 1·69	0·442
Race						
White (ref)	801	43·8	0·148	1·00	–	
Black	59	49·1		0·97	0·59, 1·58	0·89
Hispanic	206	52·0		1·36	0·97, 1·92	0·075
Other	97	50·6		1·24	0·76, 2·03	0·391
Marital status						
Single	693	52·8	<0·0001	1·57	1·20, 2·05	0·001
Married (ref)	479	40·8		1·00	–	
Highest education						
High school or less	459	51·7	<0·0001	1·48	1·07, 2·05	0·018
Some college	432	49·2		1·76	1·30, 2·40	0·0003
College graduate (ref)	287	33·8		1·00	–	
Current smoker						
Yes	240	59·1	0·0002	1·47	1·03, 2·10	0·034
No (ref)	931	44·7		1·00	–	
BMI category (kg/m^2^)						
<25 (ref)	341	42·5	0·035	1·00	–	
25 to <30	439	47·7		1·09	0·81, 1·48	0·570
≥30	331	52·4		1·25	0·89, 1·74	0·196
Poor mental health (>14 d)						
Yes	170	60·7	0·001	1·63	1·06, 2·50	0·027
No (ref)	989	45·3		1·00	–	
Heavy drinker						
Yes	1071	46·9	0·874	0·91	0·52, 1·59	0·742
No (ref)	83	47·8		1·00	–	
Leisure-time physical inactivity						
Yes	422	59·6	<0·0001	1·85	1·37, 2·50	<0·0001
No (ref)	756	42·2		1·00	–	
Number of ACE						
0 (ref)	380	42·0	0·071	1·00	–	
1	261	49·4		1·25	0·89, 1·74	0·199
2	162	49·5		1·07	0·70, 1·63	0·750
3+	376	50·7		1·42	1·02, 2·00	0·041

* Consumed fruits, dark green vegetables and other vegetables less than twice a day.

† Weighted logistic model includes ACE, gender, age, race, marital status, education, BMI, smoking status, mental health status, alcohol use and leisure-time physical inactivity.

Table 3 shows the weighted prevalence of low fruit and vegetable intake as well as AOR for each of the ACE types. Participants with a low intake of fruits and vegetables were more likely to report parental divorce/separation (52·5 *v*. 44·6 %) and physical abuse (54·9 *v*. 45·2 %). In the adjusted model, the odds of low fruit and vegetable intake were significantly higher among the participants who reported parental divorce/separation compared to those who did not experience this particular type of ACE (AOR 1·50, 95 % CI 1·13, 1·98).

**Table 3:** Weighted prevalence and adjusted OR of low fruit and vegetable intake by each type of adverse childhood experiences (ACE), Nevada Behavioral Risk Factor Surveillance System 2017, adults ≥18 years (*n* 2939)

Low intake of fruits and vegetables*						
Types of ACE	*n*	%	*P*-value	Adjusted OR†	95 % CI	*P*-value
Lived with someone who was mentally ill						
Yes	339	45·7	0·504	0·99	0·67, 1·44	0·939
No	992	47·2		1·00	–	
Lived with a substance abuser						
Yes	143	45·6	0·642	0·87	0·64, 1·19	0·165
No	914	45·2		1·00	–	
Family member incarceration						
Yes	95	45·1	0·625	0·64	0·39, 1·06	0·082
No	1095	47·5		1·00	–	
Parents divorced/separated						
Yes	409	52·5	0·009	1·50	1·13, 1·98	0·005
No	781	44·6		1·00	–	
Interpersonal violence						
Yes	226	50·0	0·363	0·99	0·68, 1·44	0·964
No	964	46·6		1·00	–	
Physical abuse						
Yes	276	54·9	0·006	1·22	0·85, 1·76	0·282
No	914	45·2		1·00	–	
Verbal abuse						
Yes	420	50·3	0·129	1·13	0·82, 1·54	0·456
No	770	45·8		1·00	–	
Sexual abuse						
Yes	150	41·8	0·136	0·75	0·64, 1·19	0·165
No	1040	48·0		1·00	–	

* Consumed fruits, dark green vegetables and other vegetables less than twice a day.

† Weighted logistic model includes ACE, gender, age, race, marital status, education, BMI, smoking status, mental health status, alcohol use and leisure-time physical inactivity.

## Discussion

ACE has been linked to risky health behaviours, chronic health conditions, low life potential and early death^(1–5,23,24)^. Using a population-based study design, the study aimed to examine the relationships between ACE and low intake of fruits and vegetables in adulthood. Our study extends the literature by commenting on the impact of ACE on dietary behaviour in adulthood, which is a potential intermediate outcome for common chronic diseases. With approximately three in every five adults experiencing at least one ACE as a child, the prevalence of ACE in our study is comparable to that of previous population-based studies^(6)^. Overall, our findings establish that the odds of low fruit and vegetable intake were significantly higher among those who experienced three or more types of ACE. However, contrary to our study hypotheses, we did not find a graded relationship between the number of ACE and low intake of fruits and vegetables. When individual ACE types were examined, exposure to parental divorce/separation was the only ACE type associated with a low intake of fruits and vegetables.

Few previous studies corroborate the relationships between ACE and dietary behaviour in adulthood found in this study. Russel *et al*.^(25)^ conducted a cross-sectional study among adults in England and found that adults with unhappy and violent childhood were at a higher risk for low daily fruit and vegetable intake (AOR 2·67, 95 % CI 2·15, 3·06) compared to those with happy and non-violent childhoods. The OR of low fruit and vegetable intake reported in that study was significantly higher than one found in our study. This may be due, in part, to the different measures used in our study *v*. a previous study, in which self-assessment of childhood experience was gauged by asking two 10-point-scale questions. Another study conducted among college students has found that higher ACE scores were associated with poorer lifestyle habits, including lower fruit and vegetable intake^(26)^. One of the potential explanations for these observed associations between ACE and fruit and vegetable intake could be that adults with multiple types of ACE are exposed to toxic stress, leading them to use food as a coping mechanism^(27,28)^. People who are exposed to stress tend to have an increased drive to eat highly palatable, comfort food (i.e. high calorie-dense foods with a high content of sugar and fat) while decreasing their consumption of healthy foods, including vegetables and fruits^(29,30)^.

Another highlight of this study is that exposure to parental divorce/separation is a significant predictor of low intake of fruits and vegetables when individual ACE types were examined. Exposure to parental divorce/separation in childhood has been linked previously to poorer physical and mental health in adulthood and to stress biomarkers such as cortisol and C-reactive protein^(31,32)^. A population-based study in Greece reported that parental divorce is significantly associated with overweight among children^(33)^. Because parental divorce/separation entails significant alterations in socioeconomical status and family environment surrounding a child, this particular types of ACE may have a consistent and lasting impact on dietary behaviour^(34)^.

Several theories consistent with our findings connect ACE with the dietary behaviour in adulthood^(10,35)^. There is increasing evidence suggesting that exposure to toxic stress during childhood might cause altered brain development leading to an array of negative consequences. These include impacts on the plastic processes of brain development, emotional regulation, cognitive response, memory and learning, and autonomic, endocrine and immune systems^(10)^. Exposure to ACE during critical development period has a long-term effect on CNS areas associated with cognitive function, such as decision-making and motivated behaviour^(36)^. Early stress may lead one to develop poor self-regulation skills, creating a proclivity for unhealthy lifestyle choices, including unhealthy diet^(10)^.

The study also identified that both sociodemographic factors and health behaviours play significant roles in relationships between ACE and low fruit and vegetable intake. In terms of sociodemographic factors, being male, single and having less than college education had an increased risk for low fruit and vegetable intake. The odds of consuming low fruits and vegetables were also higher among those who were current smokers, had poor mental health status and did not engage in leisure-time physical activity. Findings are consistent with earlier works demonstrating that gender, marital status, education level as well as smoking status, mental health status and physical activity level are significant predictors of fruit and vegetable consumption^(15,35,37,38)^. Future analyses are needed to explore the potential interactions between these variables. In addition, further investigation is needed to determine if other components of dietary behaviour, such as the consumption of high-fat, high-calorie diet, could impact the strength of association between fruit and vegetable consumption among adults with ACE.

There are some strengths and limitations to our study that should be kept in mind when interpreting our findings. The primary strength of this study is a population-based study design with a large sample size. This is one of the first studies, to our knowledge, to examine the relationships between cumulative ACE scores and fruit and vegetable intake. In addition, the study further explored the impact of individual ACE measures on fruit and vegetable intake in adulthood. The results of this study should be interpreted in light of several considerations. It is known that a number of ACE cluster together among subgroups of populations, and an examination of individual ACE might overestimate the effect by the presence of other ACE^(4,39)^. This study is limited by the cross-sectional design, and as such, it precludes making an analysis of the temporal relationship between ACE and low fruit and vegetable intake. Second, we relied on retrospective self-reports of ACE and fruit and vegetable intake, which may be affected by recall or other reporting bias. Longitudinal studies of child abuse demonstrate that adults may underreport incidences of child abuse when reporting on their experience as an adult^(40,41)^. The accuracy of dietary surveys relies on respondents’ memory, and respondents may adjust their reported consumption patterns to simplify the interview process as well as to impress the interviewer^(42,43)^. We asked how many times per day, week or month the participants consumed fruits and vegetables, which did not account for serving size, nor were any questions asked about other food groups. Socioeconomic status is known to affect fruit and vegetable intake among adults. However, income level was not included in the analysis due to a large missing value. Instead, education level was included in the analysis. Lastly, the measurement of ACE omits other early-life experiences such as childhood socioeconomic status, food insecurity, emotional and physical neglect, which may also have a lasting impact on dietary behaviour in adulthood.

## Conclusion

Despite certain limitations, findings of this study are noteworthy. The study suggests that the cumulative effects of experiencing multiple ACE and a specific ACE, notably exposure to parental divorce/separation, deteriorate fruit and vegetable intake in adulthood. The growing evidence connecting ACE scores with high-risk behaviour and the relatively high prevalence of ACE in the United States highlight the need for targeted public health interventions and policies to prevent child abuse and household dysfunction among high-risk populations. In addition, it is equally important to promote fruit and vegetable intake among the populations experiencing ACE through appropriate strategies. Future research should attempt to examine how ACE translates into dietary behaviour longitudinally and explore ways to promote healthy eating among those with ACE.
